# Deciphering the regulatory role of ELF5 in buffalo lactation

**DOI:** 10.3389/fvets.2025.1662345

**Published:** 2025-10-06

**Authors:** Ruixia Gao, Rongping Wang, Lige Huang, Xinyang Fan, Lindong Qian, Yongwang Miao

**Affiliations:** ^1^Faculty of Animal Science and Technology, Yunnan Agricultural University, Kunming, Yunnan, China; ^2^Institute of Animal Genetics and Breeding, Yunnan Agricultural University, Kunming, Yunnan, China; ^3^Faculty of Animal Husbandry and Veterinary Medicine, Yunnan Vocational and Technical College of Agriculture, Kunming, Yunnan, China

**Keywords:** buffalo, *ELF5* gene, overexpression and knockdown, gene function, milk protein synthesis

## Abstract

E74 Like ETS Transcription Factor 5 (ELF5) has been implicated in milk protein synthesis in various mammals, but its precise roles and mechanisms in buffalo have remained largely unknown. This study successfully isolated and characterized *ELF5* from buffalo mammary gland tissues, revealing a 768 bp coding sequence (CDS) that translates into a 255 amino acid protein. Bioinformatics analysis identified a conserved ETS domain within ELF5, crucial for transcriptional regulation, along with several predicted post-translational modification sites, including phosphorylation, N-glycosylation, and N-myristoylation. Molecular docking analysis further showed that ELF5 probably interacts with the STAT5A protein through hydrogen bonds and salt bridges, and forms hydrogen bonds with STAT5B, suggesting potential regulatory interactions with STAT5A and STAT5B. Experimentally, ELF5 was localized to the nucleus and cytoplasm of buffalo mammary epithelial cells (BuMECs). Notably, *ELF5* expression was highest in the buffalo mammary gland among the eight tissues and was significantly higher in lactating BuMECs than in non-lactating BuMECs. Functionally, in the BuMECs, overexpression of *ELF5* significantly upregulated mRNA and protein levels associated with milk protein synthesis, increased casein concentrations, and enhanced BuMECs metabolic activity associated with proliferation. These effects were mediated through the JAK2-STAT5 and PI3K/AKT1/mTOR signaling pathways. Conversely, *ELF5* knockdown led to the opposite effects. Collectively, these findings provide novel insights into the molecular mechanisms of *ELF5*-mediated regulation of milk protein synthesis in buffalo, highlighting its potential as a key factor in enhancing milk production.

## 1 Introduction

Milk is a highly nutritious food, providing essential components like lactose, lipids, proteins, minerals, and water crucial for growth and development (1). Milk proteins are derived from various sources, including blood and cells, and are primarily produced by secretory cells in the mammary glands. This intricate process is genetically regulated: proteins are synthesized in the rough endoplasmic reticulum (RER), undergo post-translational modifications in the Golgi apparatus and secretory vesicles, and are then released into the mammary gland lumen (2). Mammary epithelial cells (MECs) are the functional units of lactation in dairy cows, and their proliferation and milk protein synthesis capabilities directly influence mammary gland development and milk production. Buffalo milk is world-renowned for its excellent quality, rich in essential amino acids such as leucine, lysine, and valine, making it particularly suitable for cheese production (3). The Binglangjiang buffalo, a river buffalo native to western Yunnan Province, China, stands out with its high milk protein content (4.60%) (4, 5). This makes it an ideal model for studying milk production. Consequently, investigating the regulatory mechanisms of milk protein synthesis in buffalo mammary epithelial cells (BuMECs) is vital for enhancing buffalo milk quality.

Milk quality is a complex quantitative trait with moderate heritability, influenced by multiple genes (6, 7). Among these, E74 like ETS transcription factor 5 (ELF5), a member of the E-twenty six transformation-specific (ETS) family, plays a pivotal role in mammary gland development during lactation (8). ELF5 is epithelial-specific and features an N-terminal Pointed (PNT) domain—an 83-amino acid segment with strong transactivation activity, resembling a sterile alpha motif (SAM) domain. This structural characteristic hints at its regulatory significance within mammary epithelial cells (9–11). However, *ELF5* transcript variants exhibit differential expression across various tissues (12, 13). For instance, in humans, one transcript variant is found in normal breast tissue, while others are associated with breast cancer samples (9). In mice, two *ELF5* transcript variants have been identified, but only one transcript variant (NM_010125) is in mammary gland (14). Similarly, cattle have two reported transcript variants in the NCBI database, with one (AF049702, 768 bp) being extensively studied (15). Although three *ELF5* transcript variants have been reported in buffalo (accession no: XM_044929271.2, XM_025266242.3, XM_006062381.4), the specific *ELF5* transcript variant present in buffalo mammary glands remains uncharacterized.

Recent studies have increasingly linked *ELF5* to the regulation of milk protein gene expression. For example, ELF5 has been shown to transactivate the Whey Acidic Protein (WAP) promoter *in vitro* (16). Furthermore, heterozygous *ELF5* knockout mice (*ELF5*^+/−^) exhibit mammary alveolar hypoplasia and reduced milk production during pregnancy (8). Its importance is further underscored by observations in prolactin receptor knockout mice (*PRLR*^−/−^), where *ELF5* re-expression rescued alveolar morphogenesis, demonstrating its compensatory role in mammary gland development (15). In goats, a significant association between the g.27147C > G substitution and milk protein yield has also been reported (17). Despite these findings, the precise role of *ELF5* in buffalo mammary glands and its impact on lactation remain unclear. Our study aims to address this gap by isolating and characterizing *ELF5* transcript variants in buffalo mammary gland tissues. We will also explore the function and molecular mechanisms through which *ELF5* regulates milk protein synthesis in BuMECs. The insights gained from this research will significantly deepen our understanding of *ELF5*’s regulatory role in milk protein synthesis, providing a scientific foundation for improving dairy production.

## 2 Materials and methods

### 2.1 Sample collection and ethical approval

Sample collection was conducted in accordance with the Guide for Animal Care and Use of Experimental Animals and approved by Yunnan Agricultural University (No. YNAU2019llwyh019). Tissue samples, including the heart, liver, spleen, lung, kidney, brain, mammary gland and ovary, were collected from five lactating (60 days postpartum) and five non-lactating (60 days pre-partum), 4-year-old, third-parity female Binglangjiang buffalo (river type). Samples were obtained using a puncture technique, as described by Wani (18). All buffalo were maintained under consistent feeding and management conditions.

### 2.2 Cloning and identification

Total RNA was isolated from tissues and BuMECs using RNAiso Plus (TaKaRa, Dalian, China). The RNA quality was assessed via agarose gel electrophoresis and quantified using a NanoDrop 2000 UV–Vis spectrophotometer (Thermo Fisher Scientific, United States). Complementary DNA (cDNA) synthesis was performed using the M-MLV reverse transcription kit (TaKaRa, Dalian, China), with the resulting cDNA diluted to 300 ng/μL and stored at −80 °C. For cloning and identification of *ELF5* transcript variants, the primers were designed based on the reference sequences XM_044929271.2 and XM_025266242.3 for *ELF5* amplification (Supplementary Table S1). PCR was performed according to the manufacturer’s instructions for 2×Es Taq Master Mix (CWBIO, Beijing, China). The PCR protocol for *ELF5*_XM_044929271.2 included an initial denaturation at 95 °C for 3 min, followed by 35 cycles of denaturation at 95 °C for 40 s, annealing at 56 °C for 30 s, and extension at 72 °C for 40 s, with a final extension at 72 °C for 5 min. For *ELF5*_XM_025266242.3, the only difference was the annealing temperature of 58.5 °C. PCR products were detected via 1.0% agarose gel electrophoresis. Purified PCR products were ligated into the pMD18-T vector (TaKaRa, Dalian, China) at 4 °C for 16 h. The ligated products were transformed into DH5α competent cells, and 10 monoclonal colonies were selected for PCR and bidirectional sequencing by Shanghai Sangon Biotech Co., Ltd.

### 2.3 Bioinformatics analysis

Raw sequences were processed using Lasergene 7 software package (v7.1.0) (DNAStar Inc., Madison, WI, United States). Open reading frames (ORFs) were identified using the ORF Finder (last accessed on 10 May 2025),^1^ and homologous sequences were retrieved using BLAST program in the NCBI database (accessed on 10 May 2025).^2^ Nucleotide and amino acid sequence comparisons for ELF5 were conducted using Megalign (19), with corresponding sequences information presented in Supplementary Table S2.

To investigate the structures and characteristics of ELF5, bioinformatics analyses were performed. The secondary and three-dimensional structures were predicted using SOPMA (accessed on 10 May 2025)^3^ and SWISS-MODEL (accessed on 10 May 2025),^4^ respectively. The transcriptional region structures for *ELF5* were visualized using GSDS 2.0 (accessed on 10 Jan 2025).^5^ Motif compositions and domains were analyzed using MEME suite 5.5.4 (accessed on 10 May 2025)^6^ and Batch CD-Serach Tool (accessed on 10 May 2025),^7^ with visualization of using TBtools (20). Signal peptide and transmembrane regions were predicted using SignalP-4.1 (accessed on 10 May 2025),^8^ and TMHMM-2.0 (accessed on 10 May 2025),^9^ respectively. Physicochemical characteristics and subcellular localization were predicted using ProtParam (accessed on 10 May 2025)^10^ and EukmPLoc (accessed on 10 May 2025).^11^

To elucidate the function of *ELF5* gene, functional modification sites, biological processes, molecular functions, and cellular components were predicted using PROSITE (accessed on 10 May 2025)^12^ and InterProScan (accessed on 10 May 2025).^13^ The crystal structures of ELF5, STAT5A and STAT5B were retrieved from RSCB PDB database (accessed on 10 May 2025),^14^ and the protein complexes were constructed using the ZDOCK server (accessed on 10 May 2025).^15^ Molecular docking sites between the proteins were analyzed using PDBePISA (accessed on 10 May 2025)^16^ and PyMOL software (21). Gene Ontology (GO) enrichment analysis was performed using DAVID (accessed on 10 May 2025).^17^

### 2.4 Tissue differential expression analysis

The differential expression of *ELF5* across eight tissues was assessed using real-time quantitative PCR (RT-qPCR). RT-qPCR was performed in accordance with the manufacturer’s instructions for SYBR qPCR SuperMix Plus (dib) on a QuantGene 9,600 quantitative PCR detection system (Bioer Technology, Hangzhou, China). The specific qPCR primers were designed spanning exon-exon junctions, ensuring specific amplification of the *ELF5* transcript (Supplementary Table S1). The specificity of the qPCR amplification was confirmed by sequencing the products from several tissues, all of which matched the *ELF5* sequence obtained from mammary gland tissue. *β*-actin (*ACTB*), glyceraldehyde 3-phosphate dehydrogenase (*GAPDH*) and ribosomal protein S23 (*RPS23*) served as reference genes. The geometric mean of the three 2^-ΔΔCt^ values for each gene was calculated to normalize the expression level of *ELF5* (22).

### 2.5 Construction of the *ELF5* overexpression vector

To construct the overexpression vector, specific primers containing *Hind* III and *EcoR* I restriction sites were designed (forward: 5’ AAGCTTATGTTGGACTCAGTGACACAC 3’, reverse: 5’ GAATTCTAGCTTGTCTTCCTGCCACCCAT 3’). The ELF5_EGFP overexpression plasmid was constructed using the pEGFP-N1 vector (CLONTECH Laboratories, Inc.), and it was verified through bidirectional sequencing.

### 2.6 Cell culture and transfection

To explore the function of *ELF5* at cellular level *in vitro*, BuMECs were isolated, purified and identified by our group from the mammary gland tissues of lactating Binglangjiang buffalo (60 d postpartum) (23). BuMECs were cultured in DMEM/F12 medium containing 10% fetal bovine serum (FBS, Gibco) and 2% penicillin/streptomycin/amphotericin B (Gibco) at 37 °C in a 5% CO_2_ atmosphere. When the cells reached 80% confluence, a basal culture medium containing 3 μg/mL prolactin (Sigma, St. Louis, MO, United States) was applied to induce lactation 48 h prior to cell transfection (24). Before transfection, BuMECs were starved with basic DMEM culture medium for 2 h. The pEGFP-N1 vector and negative control small interfering RNA (NC-siRNA) were utilized as negative controls for the overexpression and knockdown of *ELF5*, respectively. ELF5_EGFP (3 μg) and specific siRNA targeting *ELF5* coding sequences (siRNA_ELF5) were transfected into BuMECs in six-well plates, and the cells were harvested 48 h later for further analysis.

The information regarding primers for genes related to milk protein synthesis used in RT-qPCR is provided in Supplementary Table S1, which includes prolactin receptor (PRLR), Janus kinase 2 (JAK2), signal transducer and activator of transcription 5A (STAT5A), signal transducer and activator of transcription 5B (STAT5B), suppressor of cytokine signaling 3 (SOCS3), phosphoinositide 3-kinase (PI3K), AKT serine/threonine kinase 1 (AKT1), mammalian target of rapamycin (mTOR), beta-casein (CSN2), and kappa-casein (CSN3).

### 2.7 Subcellular localization

To determine the subcellular localization of ELF5, we performed localization experiments. Forty-eight hours after transfection with the ELF5_EGFP plasmid, mitochondria and nuclei were stained using MitoTracker^®^ Red CMXRos (Solarbio, Beijing, China) and DAPI (Solarbio, Beijing, China), respectively. ELF5 localization in BuMECs was then observed using a laser scanning confocal microscope (LSCM, Olympus, Tokyo, Japan).

### 2.8 Casein assay

To investigate the effects of *ELF5* gene overexpression and knockdown on casein levels in BuMECs, we measured the casein concentration. Following the transfection of the overexpression vector and siRNA into BuMECs for 48 h, cells were collected. The total casein concentration was determined according to the manufacturer’s instructions (Bovine Casein ELISA Kit, Mlbio, Shanghai, China).

### 2.9 Cell proliferation assay based on metabolic activity

Cell proliferation was assessed using the Cell Counting Kit-8 (CCK-8), based on the WST-8 assay. BuMECs were plated in 96-well microtiter plates at a density of 5 × 10^3^ cells per well in 100 μL and incubated at 37 °C in a 5% CO_2_ atmosphere for 24 h. Following incubation, 10 μL of CCK-8 solution (Sangon Biotech, Shanghai, China) was added, and cells were incubated for an additional 2 h at 37 °C. Absorbance was measured at 450 nm using a microplate reader (Thermo Scientific, Waltham, MA, United States) according to the manufacturer’s protocol. Three biological replicates were performed for each experiment. All data presented are the final results after this background correction has been applied.

### 2.10 Protein extraction and western blotting

The proteins of treated BuMECs were collected with Cell Lysis Buffer (Affinibody, Wuhan, Hubei, China), and the concentration of total protein was assayed using the BCA assay kit (Beyotime). The equal amounts of protein samples (approximately 30 μg of total protein) were electrophoresed in SDS-PAGE and transferred to 0.45 μm PVDF membranes (Millipore, Burlington, MA, United States). Different proteins were detected using specific primary antibodies, including rabbit anti-JAK2 (1:1,000; A80717, Nature Biosciences, Zhejiang, China), rabbit anti-JAK2 (Y1007 + Y1008; 1:1,000; A24101, Nature Biosciences, Zhejiang, China), rabbit anti-STAT5 (1:1,000; bs-1142R, Bioss, Beijing, China), rabbit anti-Phospho-STAT5 (1:1,000; bs-5619R, Bioss, Beijing, China), rabbit anti-PI3K (1:1,000; bs-10657R, Bioss, Beijing, China), rabbit anti-Phospho-PI3K (1:1,000; bs-6417R, Bioss, Beijing, China), rabbit anti-AKT1 (1:1,000; bs-0115R, Bioss, Beijing, China), rabbit anti-Phospho-AKT1 (1:1,000; bs-10133R, Bioss, Beijing, China), rabbit anti-mTOR (1:1,000; A97175, Nature Biosciences, Zhejiang, China), rabbit anti-Phospho-mTOR (S2481; 1:1,000; A27561, Nature Biosciences, Zhejiang, China), rabbit anti-*β*-casein (1:1,000; bs-0466R, Bioss, Beijing, China), and monoclonal mouse anti-β-actin (1:6,000; HC201, TransGen Biotech, Beijing, China). The membrane was incubated with the primary antibody overnight at 4 °C. The species reactivity of the antibodies is all for cow. Subsequently, the membranes were further incubated with polyclonal goat anti-rabbit IgG (1:5,000; #2491145, Millipore, United States) and polyclonal goat anti-mouse IgG (1:5,000; #2517746, Millipore). The immunoreactive bands were visualized using the supersensitive ECL detection system (Sunview, Shenzhen, China). The protein abundance was determined by Image lab (Bio-rad, California, United States).

### 2.11 Statistical analysis

All experiments included three biological replicates, and results are presented as the mean ± standard error of the mean (SEM) for each experimental group. We used a two-tailed Student’s *t*-test to assess statistical significance between two groups. Data analysis and visualization were performed using GraphPad Prism 5 software (GraphPad Software Inc., La Jolla, CA, United States). A *p*-value of < 0.05 was considered statistically significant, while *p*-values of < 0.01 and < 0.001 were regarded as highly statistically significant.

## 3 Results

### 3.1 Cloning and identification of *ELF5*

The *ELF5* gene was successfully amplified from both lactating (60 days postpartum) and non-lactating (60 days pre-partum) mammary gland tissues. Using the ORF Finder tool, the open reading frame (ORF) of the obtained sequence was identified. A homology search revealed a high degree of sequence conservation among Bovidae species, with a 97.79% sequence identity. The complete coding sequence (CDS) was determined to be 768 bp in length, encoding a protein consisting of 255 amino acid residues (Supplementary Figure S1). The identified *ELF5* CDS has been deposited in the NCBI database under the accession number KF724388.1.

**Figure 1:**
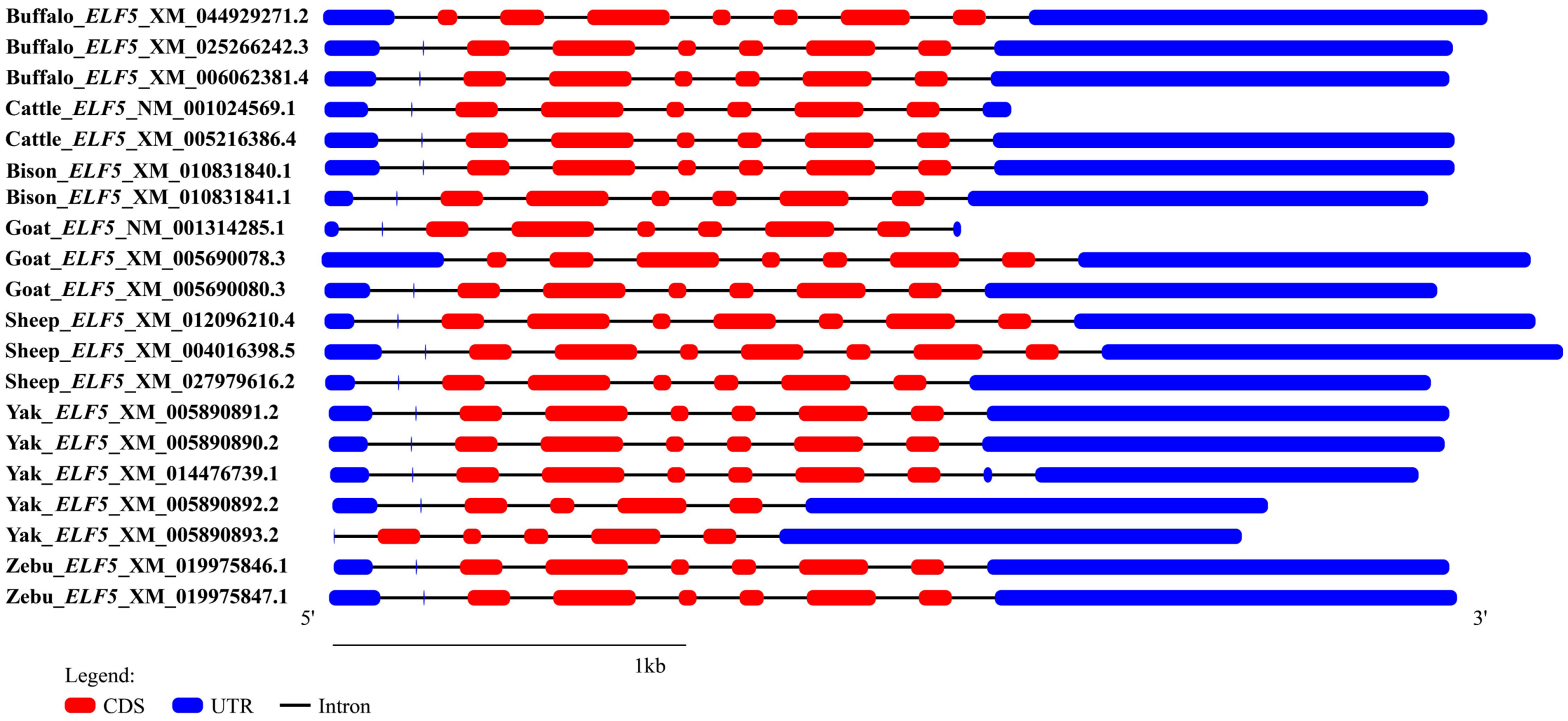
Transcriptional region structure of *ELF5* in Bovidae species.

In the buffalo mammary gland, only one transcript (*ELF5*_X2) was cloned, which aligns with the sequence XM_025266242.3 published in the NCBI database. To investigate the structural characteristics of the buffalo *ELF5* transcriptional region, we retrieved the transcript variant sequences of buffalo *ELF5*, as well as homologous sequences from other Bovidae species, from the NCBI database, and further performed reconstruction and comparative analysis of their transcriptional region structures (Figure 1). In buffalo, three transcript variants of the *ELF5* gene were identified in the NCBI database, all exhibiting variations in their 5′ untranslated regions (5′UTRs). The CDSs of *ELF5* transcript variant 2 (*ELF5_*X2, accession number: XM_025266242.3) and transcript variant 3 (*ELF5_*X3, accession number: XM_006062381.4) were identical, spanning exons 2–7, whereas transcript variant 1 (*ELF5*_X1, accession number: XM_044929271.2) encompassed exons 1–7 (Supplementary Table S2). In cattle, two transcript variants have been identified: transcript variant 1 (Cattle_X1, accession number: XM_005216386.4) and transcript variant 2 (Cattle_NM, accession number: NM_001024569.1), while both the variants share identical CDSs but have distinct 5’UTRs. In this study, only *ELF5*_X2, with a CDS length of 768 bp, was successfully isolated and characterized from buffalo mammary gland tissue. Consequently, transcript variant 2 of the *ELF5* gene (*ELF5*_X2), hereafter referred to as *ELF5*, was the focus of subsequent analyses.

### 3.2 Structural features and physicochemical characteristics of ELF5 protein

To investigate the structural similarities and differences of the ELF5 protein between buffalo and other Bovidae species, secondary and tertiary structural analyses were performed. Bioinformatics predictions revealed that buffalo ELF5 comprises 37.25% *α*- helices (95 AAs), 47.45% random coils (121 AAs), 9.41% extended chains (24 AAs), and 5.88% *β* turns (15 AAs) (Supplementary Figure S2). Analysis of the tertiary structure showed more than 99.22% sequence identity and 93.00% coverage between buffalo ELF5 and the homology modeling template (AlphaFold Protein Structure Database for cattle, template Q58DT0) (Supplementary Figure S3).

**Figure 2:**
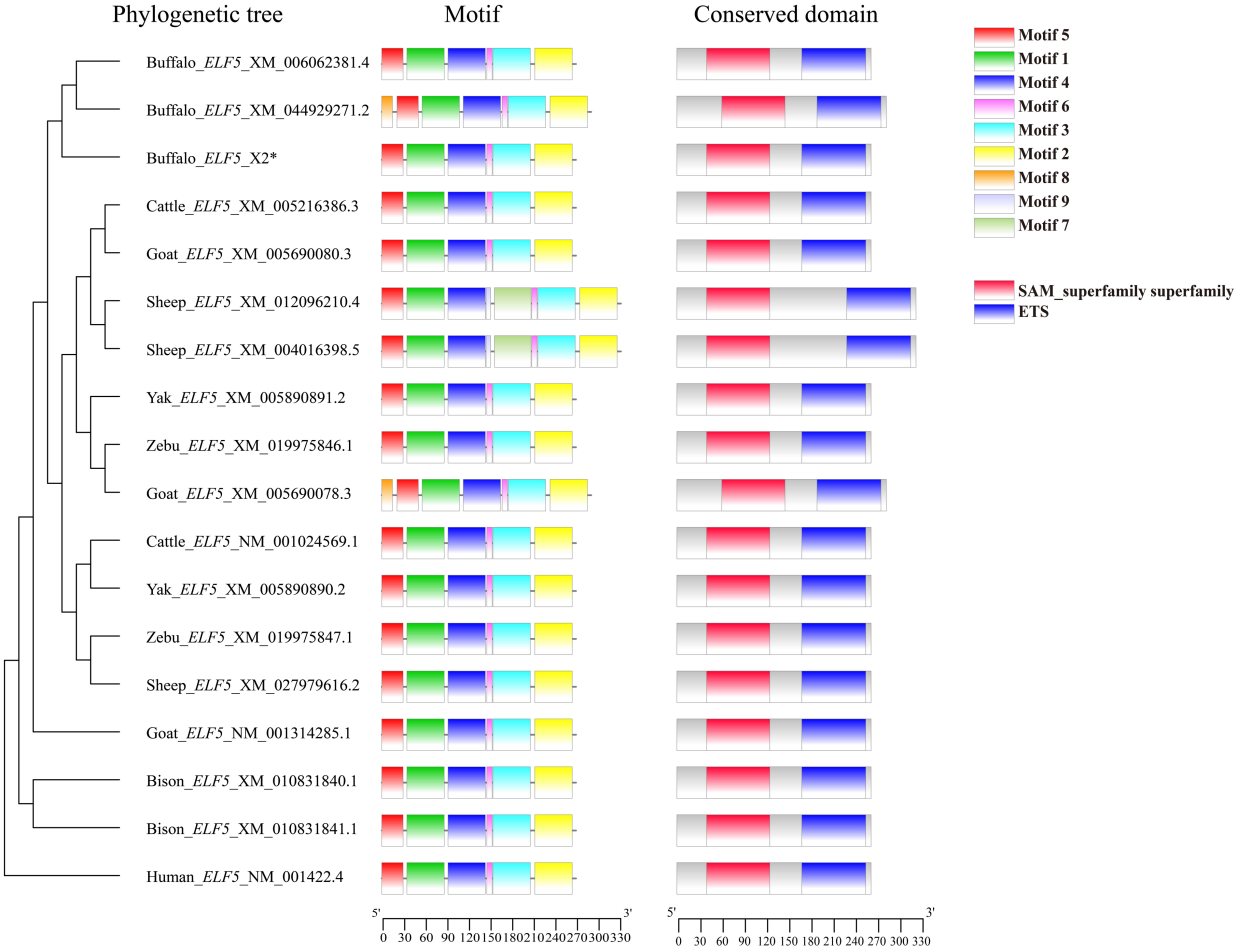
Phylogenetic tree, motif composition and conserved domain of ELF5-encoded protein. *Represents the sequence obtained in the study.

Comparisons of motifs and conserved domains further highlighted structural conservation and subtle differences of ELF5 between buffalo and other Bovidae species. Among the various transcript variants, motif 8 was unique to buffalo_*ELF5*_X1, except in goat_*ELF5*_X1. The ELF5 protein in Bovidae species harbors an ETS domain, a characteristic feature of the winged helix DNA-binding domain superfamily, as well as includes a SAM domain, which is associated with protein–protein interactions (Figure 2). Predictions suggest that buffalo ELF5 lacks both a signal peptide and a transmembrane domain (Supplementary Figure S4). Across Bovidae species, hydrophilicity was a conserved property of ELF5 proteins, with isoelectric points (pI) ranging from 5.41 to 6.30 (Supplementary Table S3). Furthermore, a phylogenetic tree constructed using amino acid sequences of ELF5 proteins revealed that buffalo forms a distinct cluster within a larger branch containing other Bovidae species, indicating the high degree of genetic conservation within Bovidae family (Figure 2).

### 3.3 Biological process, molecular function, and cellular components

To further investigate the molecular function and mechanism of *ELF5*, we performed functional predictions that *ELF5* involved in. The results revealed its involvement in several biological processes, including the regulation of DNA-templated transcription (GO:0006355), regulation of transcription by RNA polymerase II (GO:0006357), and cell differentiation (GO:0030154). Its functions were predominantly associated with sequence-specific DNA binding (GO:0043565) and DNA-binding transcription factor activity (GO:0003700). Predictions of cellular component that ELF5 was localized in the nucleus (GO:0005634), while subcellular localization analysis suggested dual localization in both the cytoplasm and nucleus (Supplementary Table S4).

### 3.4 Tissue-specific differential expression profile of *ELF5*

**Figure 3:**
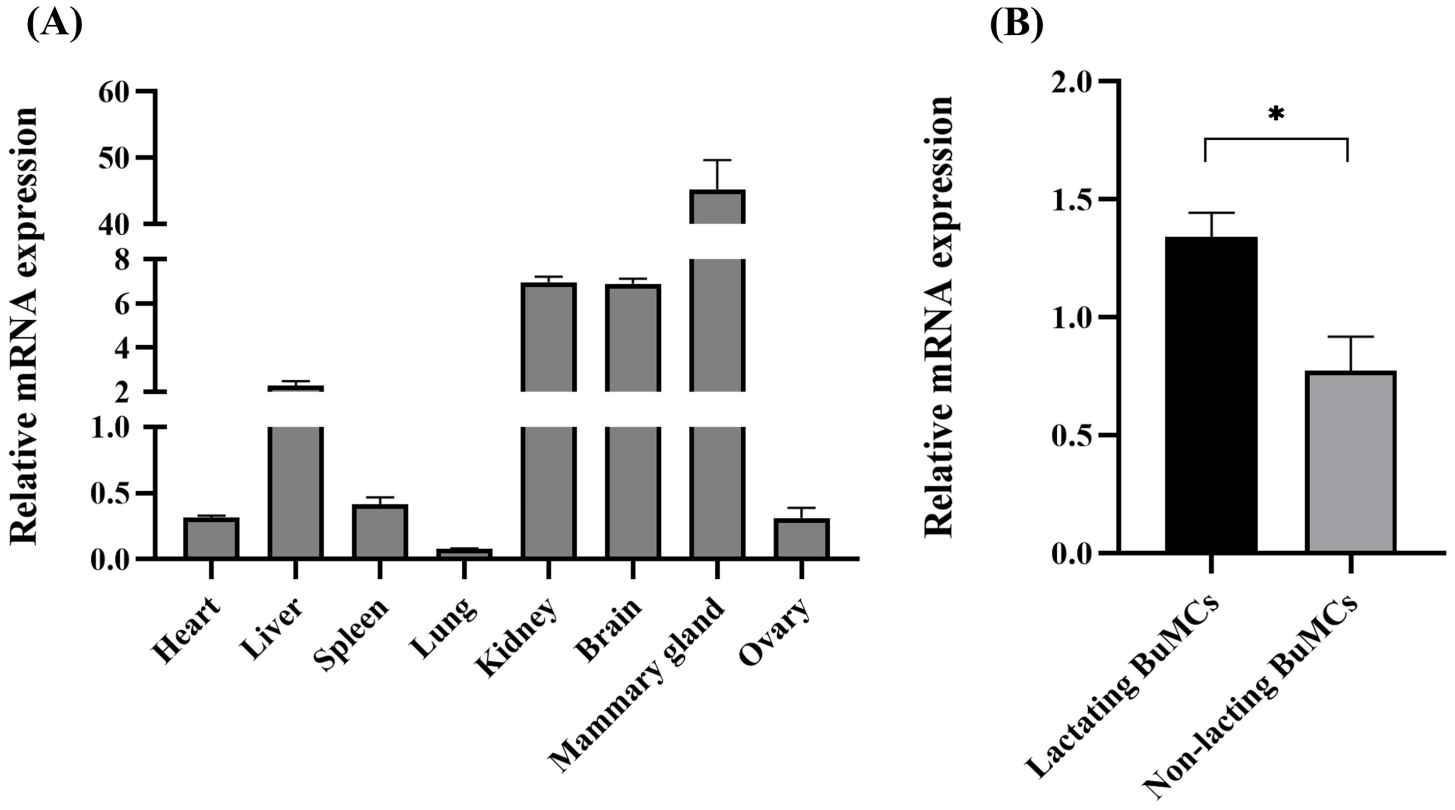
Differential expression of *ELF5* across various buffalo tissues **(A)** and in lactating versus non-lactating BuMECs **(B)**. Data are presented as means ± SEM; *p < 0.05.

With the aim of investigating the tissue-specific expression pattern of *ELF5* mRNA in lactating BLJ buffalo and gain insights into its potential function, we analyzed its differential expression across various tissues, including the heart, liver, spleen, lung, kidney, brain, mammary gland, and ovary (Figure 3A). The highest level of *ELF5* mRNA was observed in the mammary gland, followed by the brain, kidney, and liver, while the lowest expression was found in the lung. Further analysis in lactating BuMECs revealed significantly higher *ELF5* expression compared to non-lactating BuMECs, underscoring its potential role in lactation (Figure 3B).

### 3.5 Subcellular localization

**Figure 4:**
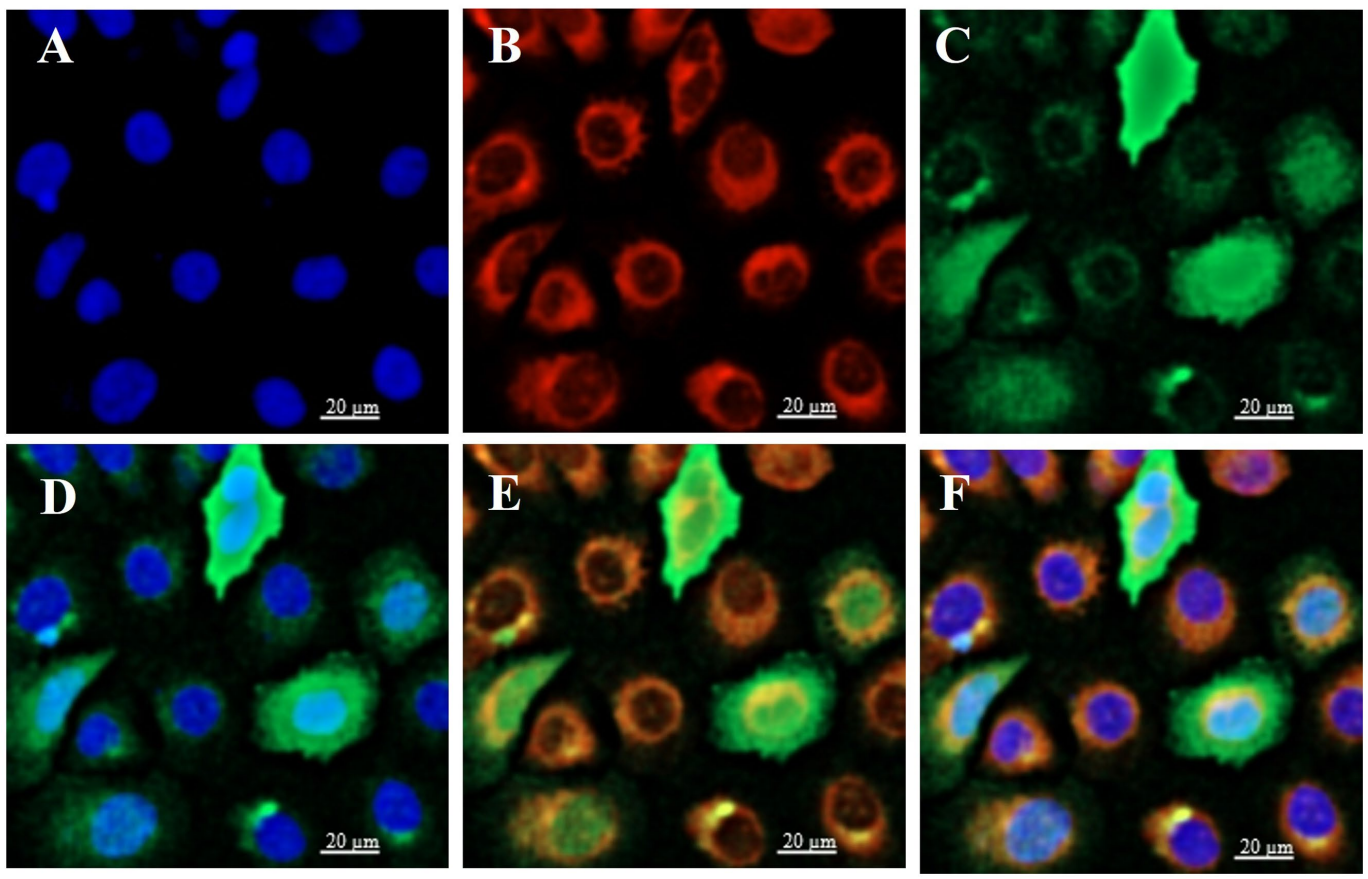
Subcellular localization of ELF5 in BuMECs, with the nucleus (blue), mitochondria (red), and *ELF5*_EGFP (green) observed via LSCM. The nucleus **(A)** and mitochondria **(B)** were stained with DAPI and MitoTracker, respectively. **(C)** Green fluorescence represents *ELF5*_EGFP expression. **(D)** Merged image of the nucleus and *ELF5*_EGFP. **(E)** Merged image of mitochondria and *ELF5_*EGFP. **(F)** Merged image of the nucleus, mitochondria and *ELF5*_EGFP. Scale bar = 20 μm.

To determine the subcellular localization of ELF5, BuMECs were transfected with *ELF5_*EGFP (Figure 4). Laser scanning confocal microscopy (LSCM) revealed that the green fluorescence overlapped with blue (Figure 4D) and red fluorescence (Figure 4E). These findings suggest that ELF5 is localized in both the nucleus and cytoplasm, aligning with the predicted subcellular localization (Supplementary Table S4).

### 3.6 Prediction of functional modification sites and molecular docking of ELF5

**Figure 5:**
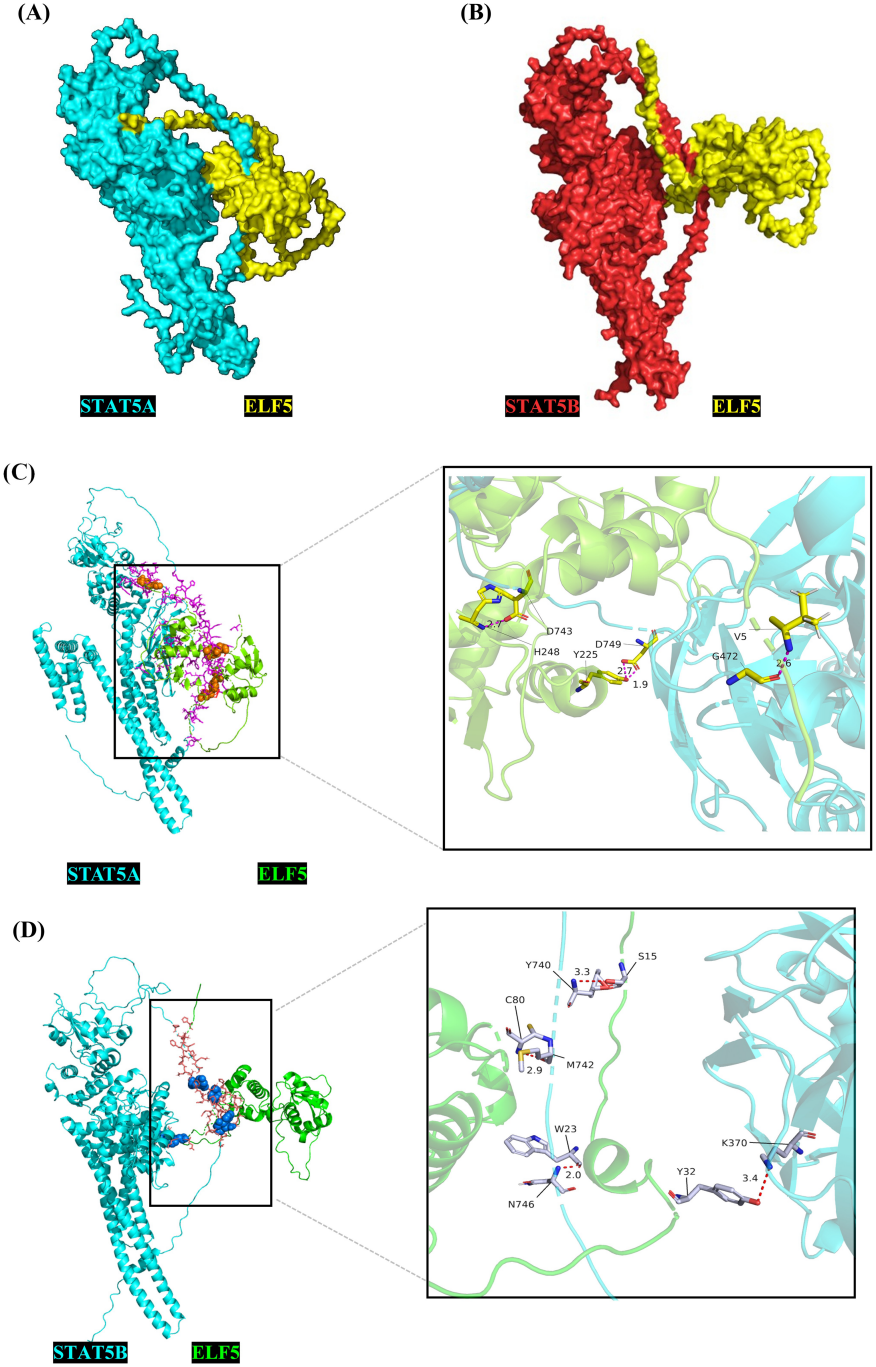
Crystal complex of STAT5A&ELF5 **(A)** and STAT5B&ELF5 **(B)** modeled using the ZDOCK server, and the molecular docking sites for STAT5A&ELF5 **(C)** and STAT5B&ELF5 **(D)** analyzed using PyMOL.

To explore the cellular functions of the buffalo ELF5 protein, we predicted its functional modification sites, including N-glycosylation sites, casein kinase II phosphorylation sites, protein kinase C phosphorylation site, N-myristoylation site and tyrosine kinase phosphorylation site 1 (Table 1). These modification sites suggest that ELF5 may be involved in critical cellular processes such as localization, transcriptional regulation and signal transduction (25, 26). Previous studies have shown that *ELF5* acts as both an upstream regulator of the *STAT5* gene by binding its promoter and as a downstream transcriptional activator of *STAT5*, indicating a regulatory feedback loop (27). Crystal structures of ELF5 (PDB ID: 1WWX), STAT5A (PDB ID: 1Y1U), and STAT5B (PDB ID: 6MBW) were retrieved from the Protein Data Bank. The complexes ELF5&STAT5A and ELF5&STAT5B were modeled (Figure 5), and the docking sites analyses revealed that ELF5&STAT5A complex is stabilized by hydrogen bonds and salt bridges, while the ELF5&STAT5B complex is primarily stabilized by hydrogen bonds (Supplementary Tables S5, S6).

**Table 1:** Predicted modification sites of ELF5 in buffalo.

Prosite	Location (amino acid)	Note
N-glycosylation site	13–16, 83–85	Asparagine
Casein kinase II phosphorylation site	15–18, 22–25, 28–31, 175–178	Phosphoserine
Casein kinase II phosphorylation site	40–43, 93–96, 132–135	Phosphothreonine
Protein kinase C phosphorylation site	55–57, 132–134	Phosphothreonine
Protein kinase C phosphorylation site	114–116	Phosphoserine
N-myristoylation site	86–91	/
Tyrosine kinase phosphorylation site 1	209–216	/

### 3.7 *ELF5* promotes milk protein synthesis via JAK2-STAT5 and PI3K/AKT1/mTOR signaling pathways, leading to increased casein concentration

**Figure 6:**
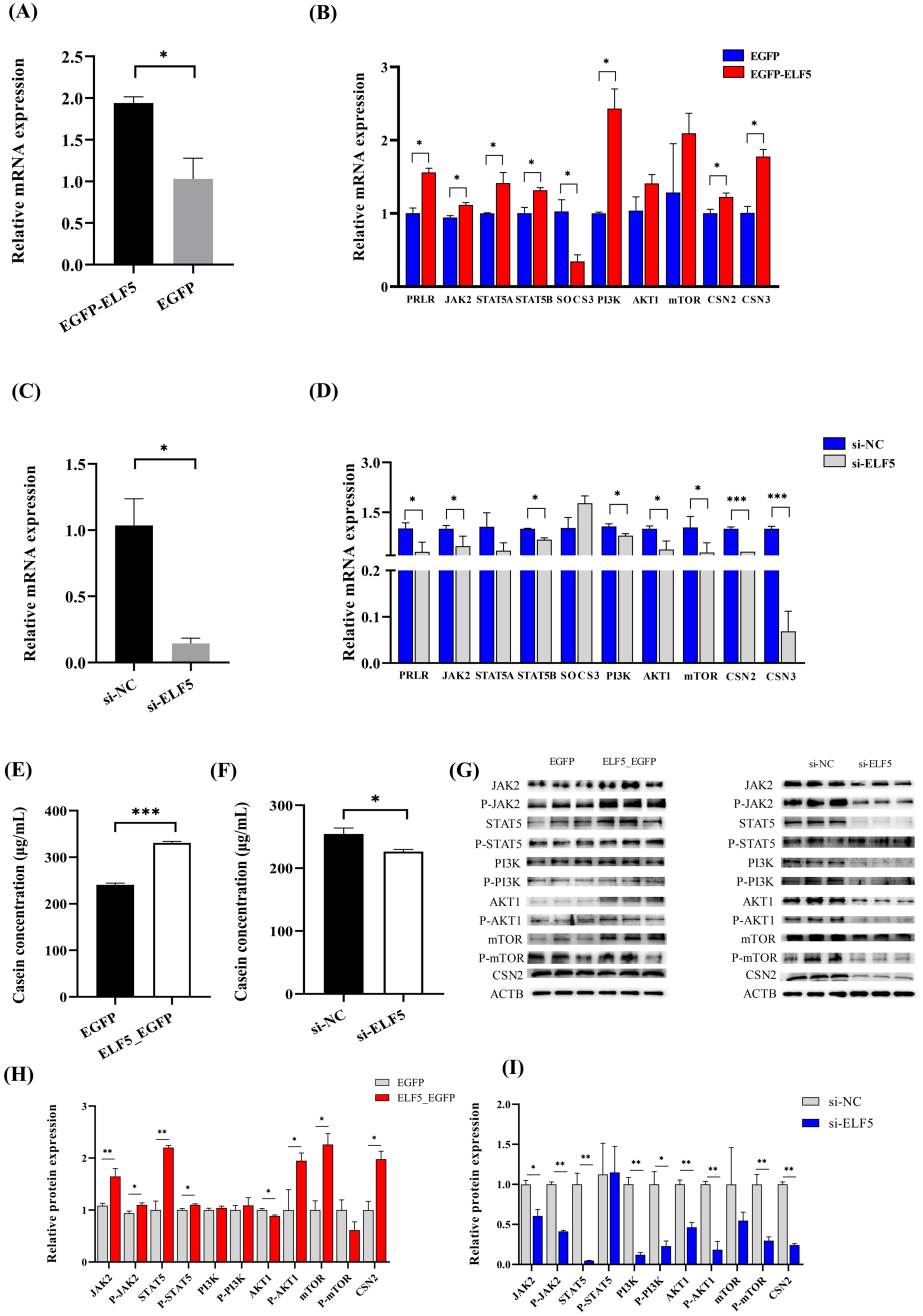
Effects of *ELF5* on milk protein synthesis in BuMECs with PRL. Changes in *ELF5* expression for overexpression **(A)** and knockdown **(C)**. Effects of *ELF5*_EGFP **(B)** and si-*ELF5* **(D)** on the expression of genes involved in milk protein synthesis within the JAK2-STAT5 and PI3K/AKT1/mTOR signaling pathways. Effects of *ELF5*_EGFP **(E)** and si-*ELF5* **(F)** on casein concentration. Effect of *ELF5*_EGFP **(G,H)** and si-*ELF5* **(G,I)** on protein levels in the JAK2-STAT5 and PI3K/AKT1/mTOR signaling pathways. Data are presented as means ± SEM from three individual cultures; *p < 0.05, **p < 0.01, ***p < 0.001.

To decipher the role of *ELF5* in BuMECs, we conducted both overexpression and knockdown experiments. For overexpression studies, we transfected BuMECs with *ELF5*_EGFP, using EGFP as a negative control. The overexpression efficiency reached 88.4% (Figure 6A). Overexpressing *ELF5* significantly increased the mRNA expression of *PRLR*, *JAK2*, *STAT5A*, and *STAT5B* (all *p* < 0.05) within the JAK2-STAT5 signaling pathway. Conversely, it downregulated *SOCS3* (*p* < 0.05) in the same pathway. In the PI3K/AKT1/mTOR pathway, *ELF5* overexpression led to a significant increase in *PI3K* mRNA expression (*p* < 0.05), while *AKT1* and *mTOR* mRNA levels remained unchanged (Figure 6B). Importantly, *ELF5* overexpression remarkably upregulated the expression of *CSN2* and *CSN3* (both *p* < 0.05), resulting in a significant increase in casein concentration (Figure 6E).

In the knockdown experiments, we achieved an *ELF5* knockdown efficiency of 85.6% (Figure 6C). Knockdown of *ELF5* significantly down-regulated the mRNA abundance of *PRLR* (*p* < 0.05), *JAK2* (*p* < 0.05) and *STAT5B* (*p* < 0.05) in the JAK2-STAT5 signaling pathway. Furthermore, it led to decreased expression of *PI3K* (*p* < 0.05), *AKT1* (*p* < 0.05), *mTOR* (*p* < 0.05), *CSN2* (*p* < 0.001), *CSN3* (*p* < 0.001) (Figure 6D), which in turn caused a significant decrease in casein concentration (Figure 6F). Western blot analysis further corroborated these findings. *ELF5* overexpression also significantly promoted the protein expression of JAK2, P-JAK2, STAT5, P-STAT5, AKT1, P-AKT1, mTOR and CSN2. Conversely, *ELF5* knockdown yielded the opposite results (Figures 6G–I; Supplementary Figures S5, S6). These findings collectively demonstrate that *ELF5* promotes milk protein synthesis through the JAK2-STAT5 and PI3K/AKT1/mTOR signaling pathways.

### 3.8 *ELF5* promotes buffalo mammary epithelial cells metabolic activity

**Figure 7:**
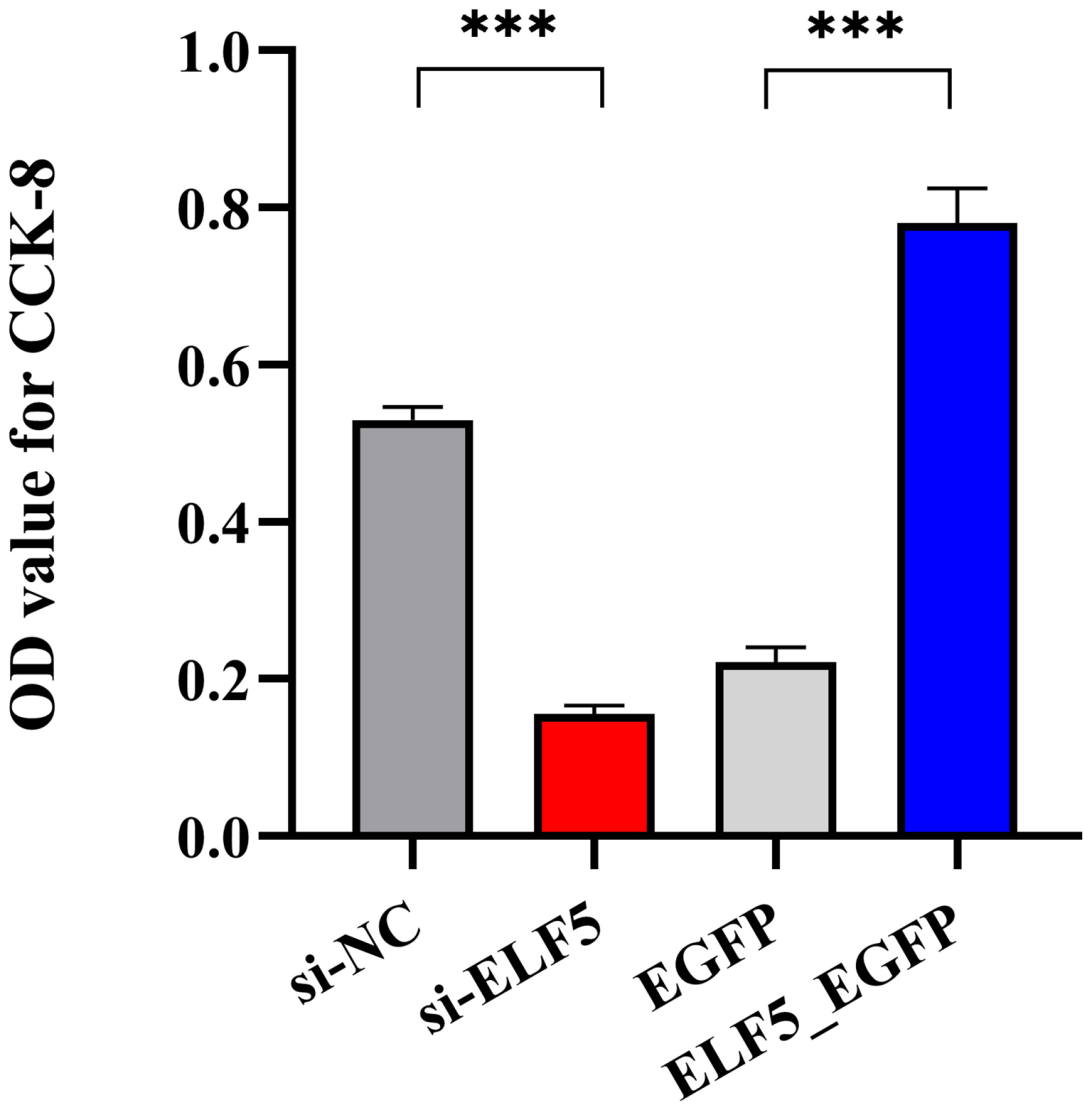
OD values of BuMECs detected using CCK-8 assays. ***P<0.001.

To delve into the role of *ELF5* in cell proliferation, we performed CCK-8 assays to assess its impact on BuMECs metabolic activity. The results demonstrated that overexpression of *ELF5* significantly enhanced BuMECs metabolic activity (*p* < 0.001), while knockdown of *ELF5* inhibited the BuMECs metabolic activity (*p* < 0.001) (Figure 7). These findings confirm that *ELF5* plays a crucial role in enhancing BuMECs metabolic activity.

## 4 Discussion

Milk is a complex biological fluid essential for nutrition, composed primarily of casein, whey protein, and triglycerides. These components are synthesized and secreted by bovine mammary epithelial cells under intricate autocrine and paracrine hormonal regulation (28). Among these, milk proteins are particularly crucial for both nutritional value and dairy production, drawing significant research attention. The transcription factor *ELF5* is highly expressed in mammary gland tissues and is known to regulate cell proliferation, differentiation, and apoptosis, playing a pivotal role in mammary gland development and function. While the role of *ELF5* in regulating milk protein synthesis has been established in other mammals, its specific function in buffalo has remained largely unexplored (29, 30).

This study addresses this gap by investigating *ELF5* in Binglangjiang buffalo, focusing on the critical time points of peak lactation (+60 days) and the dry period (−60 days). A primary finding of this work is the successful isolation and identification of a single *ELF5* transcript variant from both lactating and non-lactating buffalo mammary gland tissues. The coding sequence (CDS) of buffalo *ELF5* is 768 bp in length, encoding 255 amino acid residues. This observation is particularly significant because bioinformatics analysis of the buffalo genome, supported by entries in the NCBI database, predicts the existence of three distinct transcript variants (*ELF5*_X1, *ELF5*_X2, and *ELF5*_X3), which differ in their 5’ untranslated regions (5’UTRs). Lactation is a dynamic process, and while this study provides a critical snapshot, profiling *ELF5* expression across a broader time course, from gestation through involution, would provide a more comprehensive understanding of its dynamic regulation.

The exclusive detection of the *ELF5*_X2 in mammary tissue suggests its expression is governed by a tissue-specific promoter. The different 5’UTRs of the predicted buffalo *ELF5* transcripts imply the existence of alternative promoters at the *ELF5* gene locus. The variant identified in this study, *ELF5*_X2, which spans exons 2 to 7, is likely driven by a promoter that contains response elements for lactogenic hormones, rendering it active in mammary epithelial cells. Conversely, the promoter driving *ELF5*_X1 (encompassing exons 1–7) is probably silenced in this tissue, potentially through epigenetic mechanisms such as DNA methylation, a known regulator of *ELF5* expression in mammary epithelial cell lineages (31). This dual mechanism of selective promoter usage and epigenetic silencing ensures transcriptional precision, a hallmark of tightly controlled biological processes like lactation. This pattern of isoform expression also underscores the species-specific and tissue-specific characteristics of the *ELF5* gene, as seen in other studies where *ELF5* isoforms exhibit distinct expression profiles in tissues like the kidney and breast, or show differential localization in the blastocysts of cattle versus pigs (9, 11). Notably, the transcriptional region of buffalo *ELF5* shares high structural similarity with that of other Bovidae species and contains an ETS domain, critical for binding to specific nucleotide sequences and regulating target gene transcription (32). However, comparative genomic results revealed the differences in the 5’UTR and 3’UTR of the *ELF5* gene between buffalo, cattle, and goat, suggesting potential species-specific variations in its transcriptional regulation. These non-coding regions are known to be hotspots for regulatory evolution, containing elements that control mRNA stability, translation efficiency, and localization. This divergence in UTRs may represent a different mechanism, allowing each species to exhibit the unique ELF5 protein expression to meet distinct physiological demands, such as the high-protein, high-fat milk. In goats, transcriptomic studies have identified the *ELF5* gene as a candidate marker for milk fat synthesis and highlighted the involvement of the FoxO and MAPK signaling pathways in this process. It is inferred that *ELF5* may influence milk fat synthesis by regulating *SREBP* (33). In dairy cattle, the *ELF5* gene, acting as a downstream factor of PRL signaling and a transporter of insulin, positively regulates milk protein synthesis (34). The secondary and tertiary structures of buffalo ELF5 also mirror those of other Bovidae species. Furthermore, buffalo ELF5 is a hydrophilic protein with an isoelectric point (pI) below 7, and it lacks both a signal peptide and a transmembrane domain. These striking structural and physicochemical similarities strongly suggest that ELF5 likely shares conserved functional roles across various Bovidae species.

In the study, the differential expression results revealed that the highest *ELF5* expression in the mammary gland, followed by lower levels in the liver, kidney, and brain, and minimal expression in the heart, spleen, lung, and ovary. The high expression of *ELF5* in brain, kidney and lung indicated that *ELF5* functions in non-mammary tissues. The loss of ETS resulted in the loss of expression of some neural marker genes and the ectopic expression of the epidermal marker gene in brain precursor cells (35). In addition to their roles in normal growth and development, ETS proteins are usually involved in the formation and progression of cancer by regulating cell proliferation. Renal cancer is associated with the absence of ELF5, and the rearrangement of the *ELF5* gene has already been described in lung cancer cell lines (9). In goats, *ELF5* exhibits the high expression in the mammary gland, with lower levels in the liver, lung, kidney, and uterus, and minimal or undetectable levels in the heart, muscle, oviduct, ovary, and spleen (17), which is consistent with this study. It was reported that *ELF5* is primarily expressed in epithelial cells, suggesting its role as an epithelial-specific protein (36). Significantly, *ELF5* expression was higher in lactating BuMECs compared to non-lactating BuMECs, underscoring its functional relevance during lactation.

*ELF5* is highly expressed in tissues abundant in glandular and secretory epithelia, including the mammary gland, salivary gland, kidney, lung, and stomach. Immunohistochemical results indicated that conditional deletion of *ELF5* in the mammary glands of female mice led to defects in lobuloalveolar morphology, failure to maintain ductal characteristics typical under the non-pregnant state, and severely underdeveloped morphological maturation (29, 37). Thus, understanding ELF5’s subcellular localization is crucial for elucidating its functional mechanisms. Previous studies showed various ELF5 localization across species and cell types—predominantly nuclear in virgin mouse mammary luminal epithelial cells and mammary epithelial cells (11, 38). However, ELF5 has been observed in the cytoplasm of human breast carcinoma cells (39). In buffalo, we found that ELF5 is localized in both the nucleus and cytoplasm of BuMECs, indicating its dynamic shuttling between these compartments in response to cellular signals. In the nucleus, *ELF5* directly regulates the transcription of key milk protein genes such as *WAP* and *CSN2* (40, 41). Its cytoplasmic distribution may be influenced by post-translational modifications like phosphorylation, N-glycosylation, and N-myristoylation, or by interactions with signaling proteins like STAT5A and STAT5B (42–44). Phosphorylation regulates nuclear protein transport through multiple mechanisms. It can inhibit nuclear import by reducing the positive charge of nuclear localization signals (NLSs), thereby impairing their binding to importin proteins. Additionally, phosphorylation facilitates nuclear translocation by inducing conformational changes or disrupting nuclear export signals (NESs) (45). Specific phosphorylation sites, such as those for PKC in cytoplasmic domains, further modulate this process (42). Beyond phosphorylation, N-myristoylation, an important fatty acylation of proteins in eukaryotes, played a critical role in mitochondrial localization (46). N-glycosylation may indirectly affect the localization and transport of proteins within the cell by influencing protein folding, stability, and interactions with other molecules (47). Molecular docking analysis indirectly showed that ELF5 forms complexes with STAT5A and STAT5B via salt bridges and/or hydrogen bonds, suggesting potential protein–protein interactions within these complexes (48). Combined with the molecular docking analysis, it is inferred that the binding of ELF5 to STAT5 may induce conformational changes that expose or mask its nuclear import/export signals, ultimately affecting its subcellular localization and transcriptional activity. It should be noted that the predicted ELF5-STAT5 interaction based on molecular docking, while highly suggestive, requires further validation by co-immunoprecipitation or similar methods in future studies.

The molecular mechanisms underlying *ELF5*’s role in buffalo mammary gland development and function has not been fully elucidated. To address this, we investigated the effects of *ELF5* overexpression and knockdown on milk protein synthesis and BuMECs proliferation based on the metabolic activity, focusing on the JAK2-STAT5 and PI3K/AKT1/mTOR signaling pathways. The JAK2-STAT5 signaling pathway is paramount for mammary epithelial cell proliferation, survival, apoptosis, and milk production (49, 50). Prolactin (PRL), a critical regulator of mammary gland development and lactation, triggers intracellular signaling via its receptor (PRLR) through the JAK2-STAT5 pathway (51). PRL binding to PRLR activates JAK2, which phosphorylates STAT5A and STAT5B, leading to their nuclear translocation and subsequent regulation of milk protein-related gene transcription (52, 53). Our study showed that 3 μg/mL PRL induced lactation in BuMECs. Significantly, *ELF5* overexpression upregulated the expression of *PRLR*, *JAK2* and *STAT5B*, while downregulating *SOCS3*, a negative regulator of the JAK2-STAT5 pathway. Western blot results further confirmed that *ELF5* impacts the protein expression levels of JAK2, P-JAK2, STAT5 and P-STAT5, with *ELF5* knockdown having the opposite effect. These findings indicate that *ELF5* sensitizes BuMECs to prolactin signaling, creating a positive feedback loop that amplifies the primary lactogenic stimulus (29). Concurrently, *ELF5* was found to activate the PI3K/AKT1/mTOR pathway, which is critical for cell growth, proliferation, and overall protein synthesis capacity (54). mTOR directly regulates the protein synthesis in mammals, with AKT acting as a downstream effector kinase of PI3K (55). Our results showed that the overexpression of *ELF5* promoted *PI3K* gene expression, while *AKT1* and *mTOR* remained unchanged. *ELF5* overexpression resulted in an increase in PI3K protein, which is sufficient to activate the downstream signaling cascade via post-translational modification, leading to the phosphorylation and activation of AKT1, which in turn phosphorylates and activates mTOR. *ELF5* knockdown significantly downregulated the expression of *PI3K*, *AKT1*, and *mTOR*, significantly inhibited the protein expression of PI3K, P-PI3K, AKT1, P-AKT1, and P-mTOR, inferring that *ELF5* primarily regulates PI3K transcriptionally and mTOR post-transcriptionally. This mechanism explains a significant change in P-AKT1 and P-mTOR at the protein level while their mRNA levels remained unchanged. Intriguingly, *ELF5* overexpression promoted the expression of milk protein-related genes (such as *CSN2* and *CSN3*), increased casein concentration and CSN2 protein level, and enhanced BuMECs metabolic activity strongly associated with cell proliferation. Although the CCK-8 assay results strongly suggest a proliferative role for *ELF5*, these findings are based on metabolic activity. Future studies will employ more direct measures, such as EdU assays and cell cycle analysis via flow cytometry, to definitively confirm and further clarify its proliferative mechanisms. These findings align with studies in *ELF5* knockout mice, where *ELF5* deficiency impaired alveolar morphogenesis and prevented milk protein expression in mammary glands (11). Therefore, our study conclusively demonstrates that *ELF5* promotes milk protein synthesis, increases casein concentration, and enhances BuMECs metabolic activity through its involvement with both the JAK2-STAT5 and PI3K/AKT1/mTOR signaling pathways.

## 5 Conclusion

Our study demonstrates that *ELF5* is highly expressed in the lactating mammary gland and is dynamically localized in both the nucleus and cytoplasm of BuMECs, underscoring its crucial role in lactation. We’ve shown that *ELF5* promotes both milk protein synthesis and BuMECs proliferation assessed by metabolic activity via influencing the JAK2-STAT5 and PI3K/AKT1/mTOR signaling pathways. These findings offer valuable insights into the structure, characteristics, and functions of *ELF5*, significantly advancing our understanding of the genetic and molecular mechanisms that governs milk production traits in buffalo.

## Data Availability

The datasets presented in this study can be found in online repositories. The names of the repository/repositories and accession number(s) can be found at: https://www.ncbi.nlm.nih.gov/, KF724388.1.

## Footnotes

^1^ https://www.ncbi.nlm.nih.gov/orffinder/

^2^ https://blast.ncbi.nlm.nih.gov/Blast.cgi

^3^ https://npsa-prabi.ibcp.fr/cgi-bin/npsa_automat.pl?page=npsa%20_sopma.html

^4^ http://swissmodel.expasy.org/

^5^ http://gsds.gao-lab.org/

^6^ https://meme-suite.org/meme/tools/meme

^7^ https://www.ncbi.nlm.nih.gov/cdd/?term=

^8^ https://services.healthtech.dtu.dk/service.php?SignalP-4.1

^9^ https://services.healthtech.dtu.dk/service.php?TMHMM-2.0

^10^ http://web.expasy.org/protparam/

^11^ http://www.csbio.sjtu.edu.cn/bioinf/euk-multi/

^12^ https://prosite.expasy.org/

^13^ http://www.ebi.ac.uk/interpro/search/sequence/

^14^ https://www.rcsb.org/

^15^ https://zdock.wenglab.org/

^16^ https://www.ebi.ac.uk/pdbe/pisa/

^17^ https://david.ncifcrf.gov/

## Glossary

ACTB: *β*-actin
AA: Amino Acid
AKT1: AKT Serine/Threonine Kinase 1
BuMECs: Buffalo Mammary Epithelial Cells
CSN2: Beta-Casein
CSN3: Kappa-Casein
CCK-8: Cell Counting Kit-8
ORF: Open Reading Frame
CDS: Coding Sequence
ELF5: E74 Like ETS Transcription Factor 5
ETS: E-Twenty Six Transformation-Specific
GAPDH: Glyceraldehyde 3-Phosphate Dehydrogenase
JAK2: Janus Kinase 2
LSCM: Laser Scanning Confocal Microscope
mTOR: Mammalian Target Of Rapamycin
NC: Negative Control
pI: Isoelectric Points
PI3K: Phosphoinositide 3-Kinase
PRLR: Prolactin Receptor
RT-qPCR: Real-Time Quantitative PCR
RPS23: Ribosomal Protein S23
SAM: Sterile Alpha Motif
siRNA: Small Interfering RNA
STAT5A: Signal Transducer And Activator Of Transcription 5A
STAT5B: Signal Transducer And Activator Of Transcription 5B
SOCS3: Suppressor Of Cytokine Signaling 3
5’UTR: 5’ Untranslated Regions
