# Supplementary material for: Deciphering the regulatory role of ELF5 in buffalo lactation
Source: Front Vet Sci. 2025 Oct 6;12:1662345. doi: 10.3389/fvets.2025.1662345 (PMC12536222; doi:10.3389/fvets.2025.1662345)
Supplement: Supplementary file 1 [file Data_Sheet_1.docx]

**Figure S1.** The nucleotide sequence and the deduced amino acid sequence of buffalo_*ELF5*_X2_CDS.

**Figure S2.** The predicted secondary structure of buffalo ELF5_X2 obtained in the study. Alpha helices, extended strands and random coils are indicated with h, e and c, respectively.

**Figure S3.** Tertiary structures of ELF5_X1, ELF5 _X2, ELF5 _X3 in buffalo (A-C), ELF5_NM, ELF5_X1 in cattle (D-E), ELF5_X1, ELF5_X2 in zebu (F-G), ELF5_X1, ELF5_X2 in bison (H-I), ELF5_X1, ELF5_X2, ELF5_X3, ELF5_X4, ELF5_X5 in yak (J-N), ELF5_NM, ELF5_X1, ELF5_X2 in goat (O-Q), and ELF5_X1, ELF5_X2, ELF5_X3 in sheep (R-T).

**Figure S4.** The prediction of signal peptide (A) and transmembrane region (B) of *ELF5*_X2 in buffalo obtained in the study


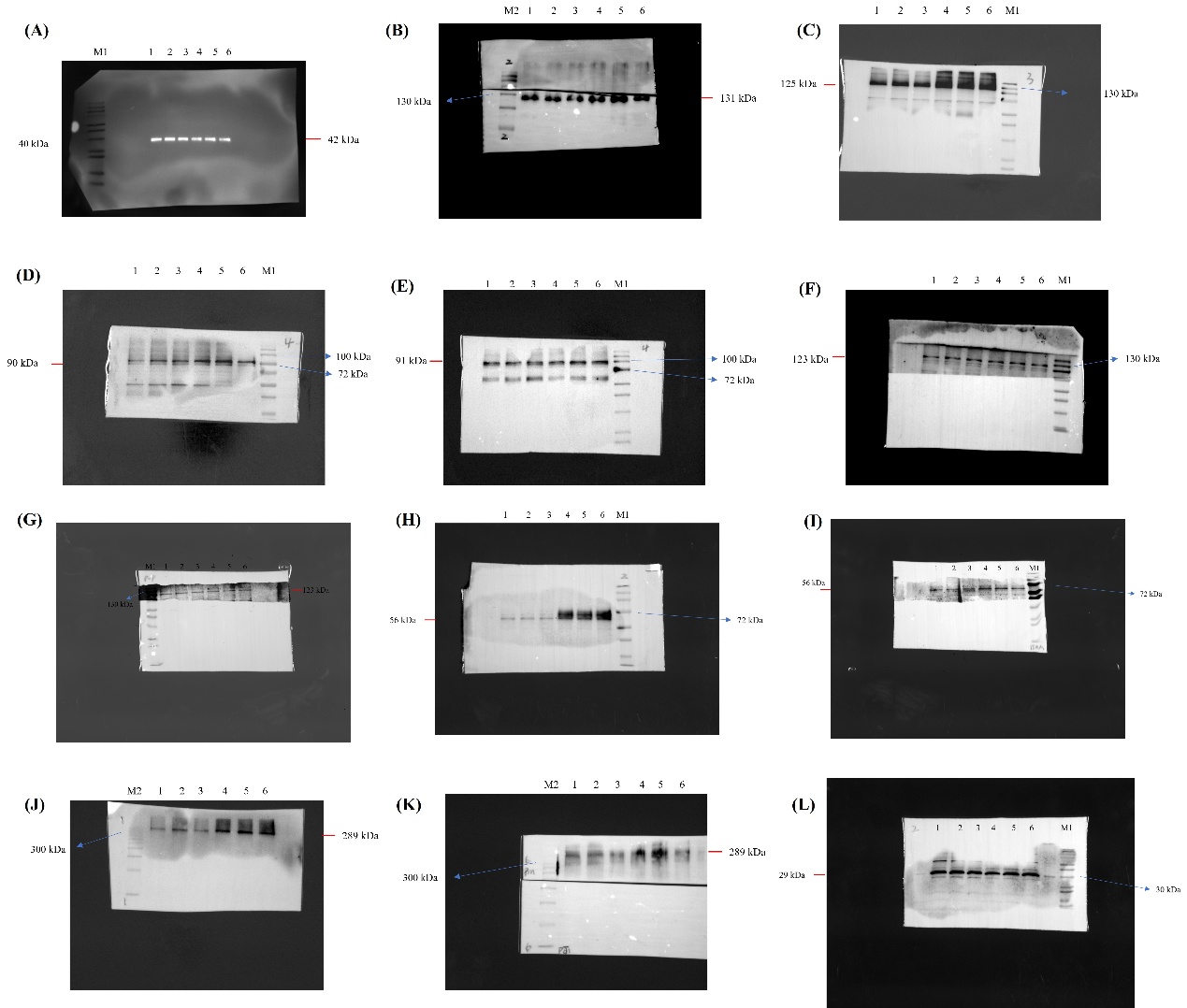


**Figure S5.** The original images for the western blotting treated with EGFP and *ELF5*_EGFP, proteins including (A) ACTB, (B) JAK2, (C) P-JAK2, (D) STAT5, (E) P-STAT5, (F) PI3K, (G) P-PI3K, (H) AKT1, (I) P-AKT1, (J) mTOR, (K) P-mTOR, (L) CSN2. Due to the small size of the gel graphs, only the corresponding marker positions have been marked. Lines 1 ~ 3: EGFP group; lines 4 ~ 6: ELF5_EGFP group; M1: 10-180 kDa tri-color prestained protein marker; M2: 25-300 kDa tri-color prestained protein marker. The right edge of membrane in figure (K) had been cropped before exposure.


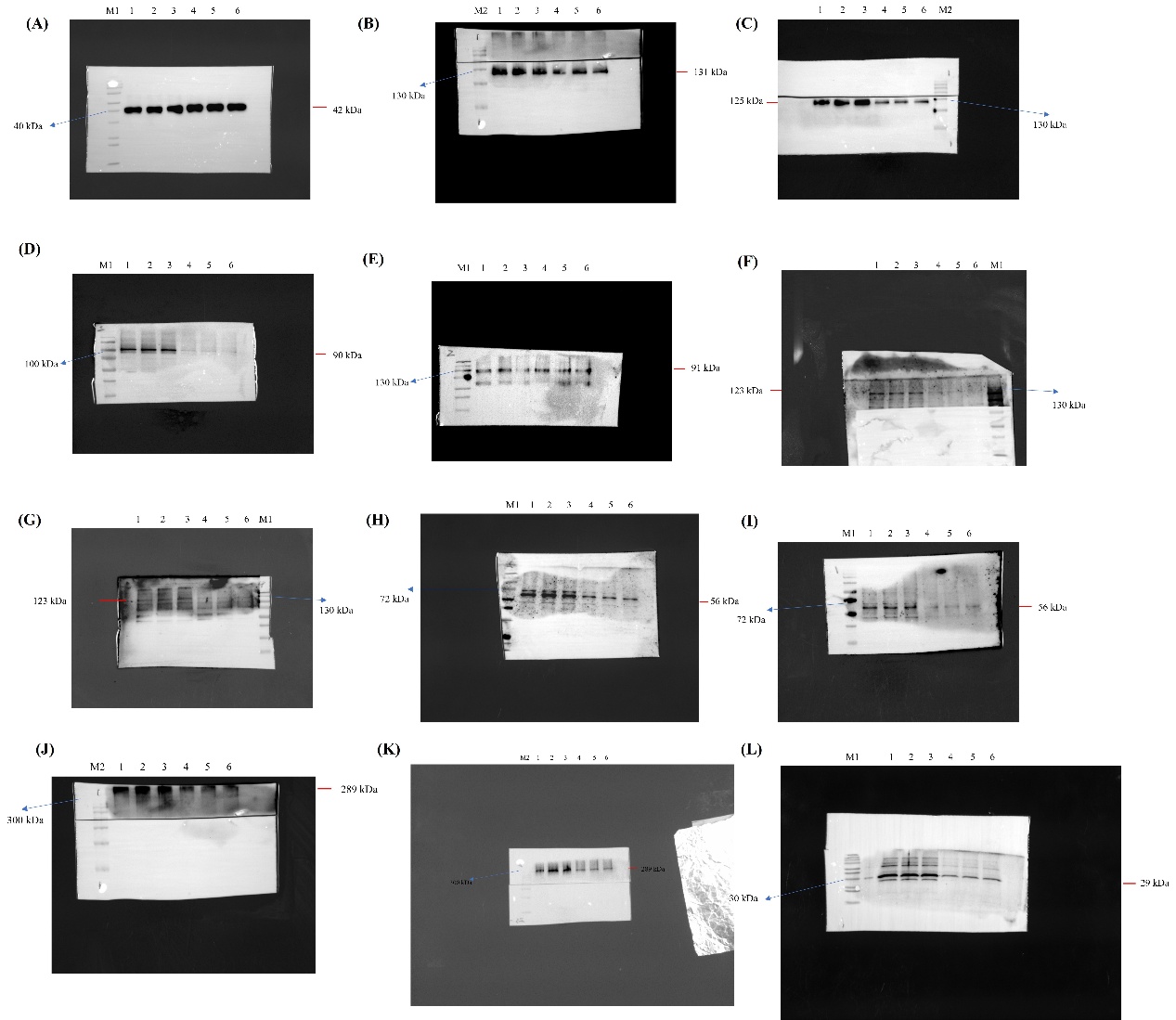


**Figure S6.** The original images for the western blotting treated with siNC and si-*ELF5*, proteins including (A) ACTB, (B) JAK2, (C) P-JAK2, (D) STAT5, (E) P-STAT5, (F) PI3K, (G) P-PI3K, (H) AKT1, (I) P-AKT1, (J) mTOR, (K) P-mTOR, (L) CSN2. Due to the small size of the gel graphs, only the corresponding marker positions have been marked. Lines 1 ~ 3: siNC group; lines 4 ~ 6: si-*ELF5* group; M1: 10-180 kDa tri-color prestained protein marker; M2: 25-300 kDa tri -color prestained protein marker. The bottom edge of membrane in figure (F) had been cropped before exposure.

**Table S1.** **Primer information using for PCR and RT-qPCR**

|  | Primers (5′ to 3′)^*^ | Product length (bp) | Annealing temperature (℃) | Usage | Efficiency |
| --- | --- | --- | --- | --- | --- |
| *ELF5* | F: ATGCGGTATCCTTGTCG  R: TGTTTGAAAAGACAGAAATCCAT | 934 | 62.8 | X1_CDS isolation, bidirectional sequencing | - |
| *ELF5* | F: CCTGTCTCTCTGACTGTGTGGA  R: CCATTACATGAGCTGGATGA | 842 | 61.0 | X2_CDS isolation, bidirectional sequencing | - |
| *ELF5* | F: CCCCTGATGTCGTGGACT  R: TGTTGAAATGGCAGAAAGAG | 196 | 53.3 | X2_Expression detection | 2.10 |
| *ELF5* | F: AAGCTTATGTTGGACTCAGTGACACAC  R:GAATTCGAATTCTAGCTTGTCTTCCTGCCACCCAT | 765 | 58.5 | Vector construction, bidirectional sequencing | - |
| *JAK2* | F: GATCTGGCAACAAGGAAT  R: CGCTGGTGGGCTTTTACT | 252 | 60.0 | Expression detection | 1.92 |
| *STAT5A* | F: TGACCGTAACCTGTGGAATCT  R: GGACCACTTGCTTGATCTGTG | 203 | 60.0 | Expression detection | 1.95 |
| *STAT5B* | F: TCCACAGGAGAACATTAAGGC  R: GCTGGAAGTCTCGCCGATGCC | 259 | 60.0 | Expression detection | 2.01 |
| *SOCS3* | F: CAGTCGGGGACCAAGAACC  R: CGAGGAGGGCGAGGAGGAG | 181 | 53.3 | Expression detection | 1.87 |
| *PRLR* | F: CCACCCACCACGACTGATGTAAA  R: ATGACAGCAGAAAGGACGG | 146 | 60 | Expression detection | 1.95 |
| *CSN2* | F: ACAGCGGCAAACAGAGGATGA  R: ACTCCCATTATTTCAGGCT | 174 | 51.5 | Expression detection | 1.85 |
| *CSN3* | F: CAATACGCTGTGAGAAAGAGG  R: TGGCAGGACTTGGCAGGCA | 244 | 62.9 | Expression detection | 1.92 |
| *PI3K* | F: CAGTCACCTCTCAACCCA  R: GCCGTAAATCATCACCAT | 160 | 60.0 | Expression detection | 1.87 |
| *AKT1* | F: CCAAGTCCCTGCTCTCGG  R: TTCGCTGTCCACCCCCTC | 266 | 60.0 | Expression detection | 2.12 |
| *mTOR* | F: ATGCTGTCCCTGGTCCTTATG  R: GGGTCAGCGAGTGGCCTTCAA | 178 | 60.0 | Expression detection | 1.98 |
| *ACTB* | F: TGGGCATGGAATCCTG  R: GGCGCGATGATCTTGAT | 196 | 60.0 | Expression detection | 1.91 |
| *GAPDH* | F: ATGGAGAAGGCTGGGGCTCA  R: GCAGGAGGCATTGCTGACAA | 144 | 60.0 | Expression detection | 1.92 |
| *RPS23* | F: GCGGCTTTGGTGACTCTA  R: CTGCCTTCCTTGGATGTG | 195 | 60.0 | Expression detection | 1.96 |

**^*^ F: Forward, R: Reverse.**

**Table S2. Nucleotide and amino acid sequence information of *ELF5* across Bovidae species**

| **Species** | **Accession number of nucleotide sequences** | **Accession number of protein sequences** | **CDS length (bp)** | **Coverage** |
| --- | --- | --- | --- | --- |
| Buffalo_X1 | XM_044929271.2 | XP_044785206.1 | 828 | exon 1-7 |
| Buffalo_X2 | XM_025266242.3 | XP_025122027.2 | 768 | exon 2-7 |
| Buffalo_X3 | XM_006062381.4 | XP_006062443.3 | 768 | exon 2-7 |
| Cattle_NM | NM_001024569.1 | NP_001019740.1 | 768 | exon 2-7 |
| Cattle_X1 | XM_005216386.4 | XP_005216443.1 | 768 | exon 2-7 |
| Zebu_X1 | XM_019975846.1 | XP_019831405.1 | 768 | exon 2-7 |
| Zebu_X2 | XM_019975847.1 | XP_019831406.1 | 768 | exon 2-7 |
| Bison_X1 | XM_010831840.1 | XP_010830142.1 | 768 | exon 2-7 |
| Bison_X2 | XM_010831841.1 | XP_010830143.1 | 768 | exon 2-7 |
| Yak_X1 | XM_005890891.2 | XP_005890953.1 | 768 | exon 2-7 |
| Yak_X2 | XM_005890890.2 | XP_005890952.1 | 768 | exon 2-7 |
| Yak_X3 | XM_014476739.1 | XP_014332225.1 | 768 | exon 2-7 |
| Yak_X4 | XM_005890892.2 | XP_005890954.1 | 483 | exon 2, exon 5-7 |
| Yak_X5 | XM_005890893.2 | XP_005890955.1 | 534 | exon 2, exon 4-7 |
| Goat_NM | NM_001314285.1 | NP_001301214.1 | 765 | exon 2-7 |
| Goat_X1 | XM_005690078.3 | XP_005690135.1 | 828 | exon 1-7 |
| Goat_X2 | XM_005690080.3 | XP_005690137.1 | 698 | exon 2-7 |
| Sheep_X1 | XM_012096210.4 | XP_011951600.1 | 945 | exon 2-4, exon 6-8 |
| Sheep_X2 | XM_004016398.5 | XP_004016447.2 | 945 | exon 2-4, exon 6-8 |
| Sheep_X3 | XM_027979616.2 | XP_027835417.1 | 768 | exon 2-7 |

| Basic physical and chemical properties | Buffalo_X1 | Buffalo_X2*X3 | Cattle_NM_X1 | Zebu_X1X2 | Yak_X1-X3 | Yak_X4 | Yak_X5 | Bison_X1X2 | Sheep_X3 | Sheep_X1X2 | Goat_NM | Goat_X1 | Goat_X2 |
| --- | --- | --- | --- | --- | --- | --- | --- | --- | --- | --- | --- | --- | --- |
| Number of amino acids | 275 | 255 | 255 | 255 | 255 | 160 | 177 | 255 | 255 | 314 | 255 | 275 | 255 |
| Molecular weight (kDa) | 32.25 | 30.06 | 30.09 | 30.09 | 30.09 | 18.87 | 20.85 | 30.08 | 30.09 | 36.62 | 30.00 | 32.18 | 30.09 |
| Isoelectric point (pI) | 5.51 | 5.61 | 5.61 | 5.61 | 5.61 | 6.30 | 6.08 | 5.61 | 5.61 | 5.50 | 5.73 | 5.41 | 5.61 |
| Instability index (II) | 54.77 | 50.42 | 50.82 | 50.82 | 50.82 | 49.34 | 45.03 | 51.87 | 50.82 | 48.38 | 50.22 | 55.29 | 50.82 |
| Grand average of hydropathicity (GRAVY) | -0.649 | -0.581 | -0.583 | -0.583 | -0.583 | -0.756 | -0.782 | -0.580 | -0.583 | -0.562 | -0.569 | -0.635 | -0.583 |
| Aliphatic index | 60.29 | 63.49 | 63.49 | 63.49 | 63.49 | 65.81 | 62.26 | 63.49 | 63.49 | 63.06 | 63.49 | 60.29 | 63.49 |

**Table S3. Physicochemical characteristics of ELF5 between buffalo and other Bovidae species**

*** represents sequence obtained in this study.**

| Location | Website | Evaluation |
| --- | --- | --- |
| Mitochondrion, Nucleus | <http://www.csbio.sjtu.edu.cn/bioinf/euk-multi/> | / |
| Cytoplasmic, Cytoplasmic and nucleus | <https://wolfpsort.hgc.jp/> | 12, 11.5 |
| Cytoplasmic, Nucleus | <https://psort.hgc.jp/form2.html> | 65.2%, 26.1% |
| Nucleus | <https://rostlab.org/services/loctree3/> | 33 |
| Nucleus, Cytoplasmic | <http://www-bs.informatik.uni-tuebingen.de/Services/MultiLoc2> | 0.78, 0.17 |

**Table S4. Predicted subcellular localization of ELF5**

**Table S5.** **Molecular docking sites of ELF5 and STAT5A proteins by PyMOL and PDBePISA**

| No. | ELF5* | STAT5A | Distance (Å) | Bond |
| --- | --- | --- | --- | --- |
| 1 | 5Val | 472Gly | 2.6 | Hydrogen bond |
| 2 | 248His | 743Asp | 2.7 | Hydrogen bond |
| 3 | 7His/NE2 | 579Glu/OE1 | 3.84 | Salt bridge |
| 4 | 248His/ND1 | 743Asp/OD1 | 3.52 | Salt bridge |
| 5 | 248His/ND1 | 743Asp/OD2 | 2.46 | Salt bridge |
| 6 | 96Glu/OE1 | 417Arg/NH1 | 3.73 | Salt bridge |

*** represents sequence obtained in this study.**

**Table S6.** **Molecular docking sites of ELF5 and STAT5B proteins by PyMOL and PDBePISA**

| No. | ELF5* | STAT5B | Distance (Å) | Bond |
| --- | --- | --- | --- | --- |
| 1 | 32Tyr | 370Lys | 3.4 | Hydrogen bond |
| 2 | 23Trp | 746Asn | 2.0 | Hydrogen bond |
| 3 | 15Ser | 740Tyr | 3.3 | Hydrogen bond |
| 4 | 80Cys | 742Met | 2.9 | Hydrogen bond |

*** represents sequence obtained in this study.**
